# Tobacco microbial screening and application in improving the quality of tobacco in different physical states

**DOI:** 10.1186/s40643-023-00651-6

**Published:** 2023-05-02

**Authors:** Ying Ning, Li-Yuan Zhang, Jing Mai, Jia-En Su, Jie-Yun Cai, Yi Chen, Yong-Lei Jiang, Ming-Jun Zhu, Bin-Bin Hu

**Affiliations:** 1 Yunnan Academy of Tobacco Agricultural Sciences, Kunming, 650021 People’s Republic of China; 2 School of Biology and Biological Engineering, Guangdong Key Laboratory of Fermentation and Enzyme Engineering, Guangzhou Higher Education Mega Center, South China University of Technology, Panyu, Guangzhou 510006 People’s Republic of China; 3 College of Life and Geographic Sciences, The Key Laboratory of Biological Resources and Ecology of Pamirs Plateau in Xinjiang Uygur Autonomous Region, The Key Laboratory of Ecology and Biological Resources in Yarkand Oasis at Colleges & Universities Under the Department of Education of Xinjiang Uygur Autonomous Region, Kashi University, Kashi, 844006 China

**Keywords:** Tobacco powder, Tobacco leaf, Microbial fermentation, Tobacco quality, Microbial community analysis

## Abstract

The first-cured tobacco contains macromolecular substances with negative impacts on tobacco products quality, and must be aged and fermented to mitigate their effects on the tobacco products quality. However, the natural fermentation takes a longer cycle with large coverage area and low economic efficiency. Microbial fermentation is a method to improve tobacco quality. The change of chemical composition of tobacco during the fermentation is often correlated with shapes of tobacco. This study aimed to investigate the effects of tobacco microorganisms on the quality of different shapes of tobacco. Specifically, *Bacillus subtilis* B1 and *Cytobacillus oceanisediminis* C4 with high protease, amylase, and cellulase were isolated from the first-cured tobacco, followed by using them for solid-state fermentation of tobacco powder (TP) and tobacco leaves (TL). Results showed that strains B1 and C4 could significantly improve the sensory quality of TP, enabling it to outperform TL in overall texture and skeleton of tobacco products during cigarette smoking. Compared with the control, microbial fermentation could increase reducing sugar; regulate protein, starch, and cellulose, reduce nicotine, improve total aroma substances, and enable the surface of fermented TP and TL to be more loose, wrinkled, and porous. Microbial community analysis indicated that strains B1 and C4 could change the native structure of microbial community in TP and TL. LEfSe analysis revealed that the potential key biomarkers in TP and TL were *Bacilli*, *Pseudonocardia, Pantoea*, and *Jeotgalicoccus*, which may have cooperative effects with other microbial taxa in improving tobacco quality. This study provides a theoretical basis for improving tobacco fermentation process for better cigarettes quality.

**Graphical Abstract:**

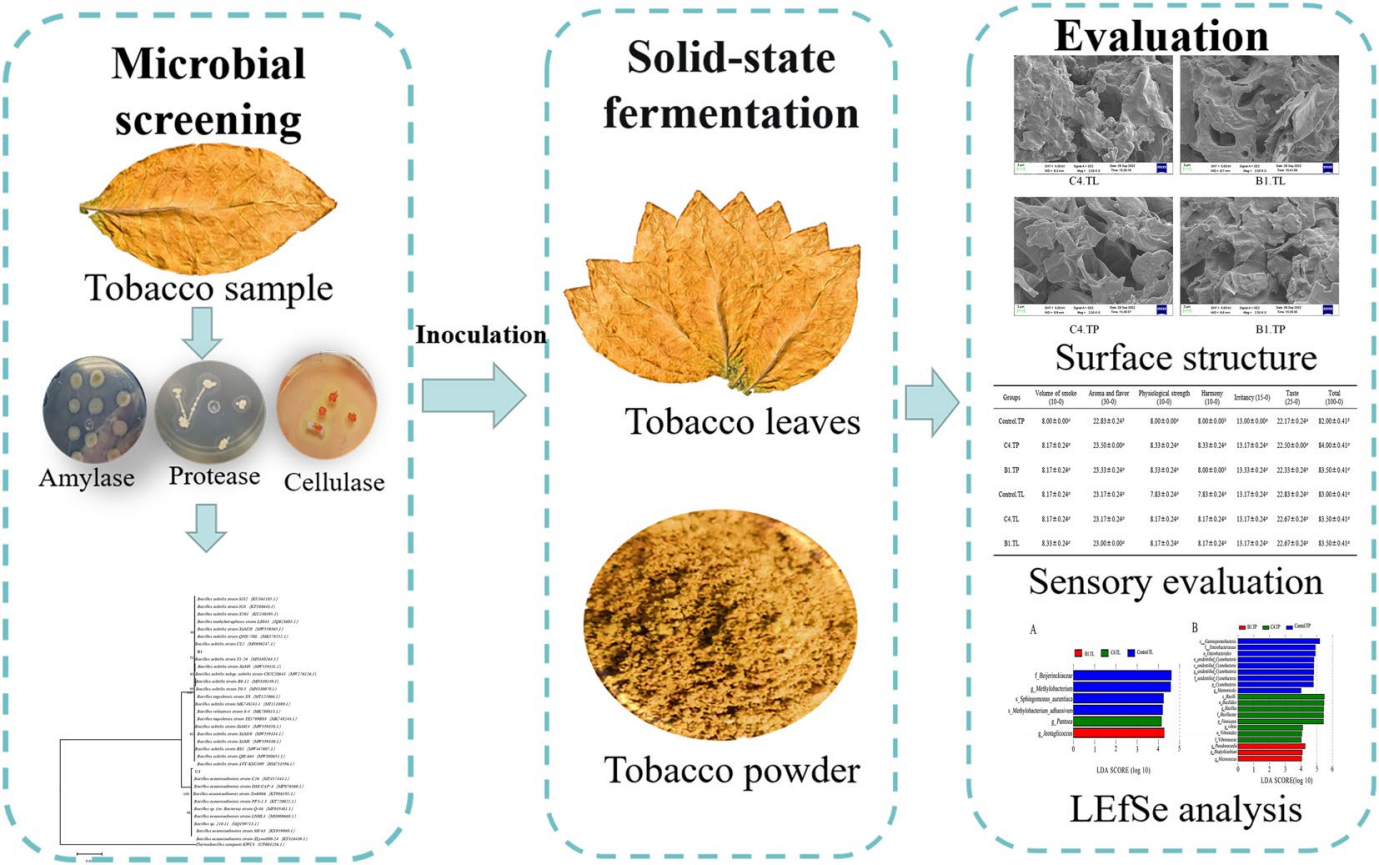

**Supplementary Information:**

The online version contains supplementary material available at 10.1186/s40643-023-00651-6.

## Introduction

Microbial fermentation plays key role in tobacco alcoholization process, which is important for improving the quality of tobacco products. Recently, more attention has been paid to the method of adding functional microorganisms, which has already been practiced in the food industry to increase food flavor substances and improve food quality. For example, microbial fermentation was reported to enhance the total aroma content and sensory quality of rice wine (Yan et al. 2019), liquor (Su et al. 2020), wine (Belda et al. 2017), soy sauce (Devanthi and Gkatzionis 2019), sauerkraut (Joyce et al. 2018), and other foods. Moreover, exogenous functional microorganisms were shown to highly reduce harmful substances (such as tobacco-specific nitrosamines (TSNA) or nicotine), shorten tobacco aging cycle, and increase tobacco total aroma content (Giovanni et al. 2007; Gong et al. 2009; Wang et al. 2004; Zheng et al. 2022). However, the adaptability of exogenous functional microorganisms in tobacco (Nawaz et al. 2022), metabolites, and metabolic pathways should be further considered in term of food safety. Interestingly, screening functional microorganisms from tobacco could improve tobacco quality during tobacco fermentation (Dai et al. 2020; English et al. 1967; Wu et al. 2022, 2021). The native microorganisms isolated from tobacco could avoid the adverse effects caused by adding exogenous microorganisms, thus playing an important role in increasing aroma content and improving tobacco quality (Liu et al. 2021; Zhang et al. 2020; Zhou et al. 2020). According to previous studies on the microbial community in tobacco showed that the dominant microorganism on the surface of tobacco was mainly *Bacillus*, which plays a key role in the degradation of macromolecules to increase aroma content and improve tobacco quality during the natural tobacco fermentation (Wang et al. 2018a). When microorganisms are screened from the surface of tobacco, their adaptability and security to grow in tobacco after inoculation and fermentation were stronger than that of exogenous inoculants. Meanwhile, few studies on the microbial interaction effects in tobacco under the condition of adding native microorganisms were reported. Thus, it is necessary to study the effect of the native microbial addition on the tobacco microbial community structure during fermentation process.

In the tobacco alcoholization process, biodegradation was also a crucial technology process (Tsuchiya et al. 2021; Wang et al. 2004). As tobacco is mainly composed of starch, cellulose, and other macromolecular substances, direct combustion will produce some harmful precursor substances, and degradation could be accompanied by the formation of aroma substances (Banožić et al. 2020). Starch, cellulose, and protein could produce a bitter, pungent, and offensive odor (Dai et al. 2020; Torikaiu et al. 2005). High contents of starch could produce an undesirable charring odor when burned. Cellulose could be degraded to form hydroxymethylfurfural (HMF) and L-glucosan at higher temperatures above 250 ℃, which has an unpleasant smell. Starch and cellulose could produce aldehydes by combustion and thermal degradation, such as formaldehyde and acetaldehyde, which are regarded as carcinogenic compounds (Banožić et al. 2020). Protein could produce the smell of charred feather and harmful substances, such as quinoline, cyanic acid and benzopyrene with cigarettes smoking (Wu et al. 2021). Starch and cellulose could be biodegraded into sugars by biological conversion. Sugar, especially reducing sugar, is an important aroma component of tobacco, which has a positive effect on tobacco-smoking characteristics, improving flavor and aroma of tobacco (Baker et al. 2005; Roemer et al. 2012; Talhout et al. 2006). Proteins can be degraded to amino acids, which could react with sugars resulted from the biodegradation of starch and cellulose, thus promoting the formation of aroma of tobacco.

At present, the industrial production of tobacco products includes the procedures of harvest, primary roasting, thresh and redrying, fermentation and alcoholization, and cigarette production. The fermentation and alcoholization are key process of cigarettes production, and most factories generally use the tobacco leaves (TL) during this process. However, each TL in the chemical composition distribution is inhomogeneity, thus making a difference in the quality of tobacco produced even in the same batch (Qin and Gong 2016; Tao et al. 2022). Few studies have been performed on the fermentation of different tobacco shapes, such as TL and TP (Pereira et al. 2016; Qin and Gong 2016). It is meaningful to explore the influence of fermentation of TL and TP on the quality of cigarette.

This study aimed to study the effects of two tobacco microbial strains on the fermentation of tobacco and comparison of TL and TP during fermentation. Specifically, two strains (*Bacillus subtilis* B1 and *Cytobacillus oceanisediminis* C4) with high amylase, cellulase, and protease activities were directly screened from the first-cured tobacco, followed by applying them in TL and TP solid-state fermentation to increase the content of aroma substances and improve the sensory quality of TL and TP products. Results indicated that after microbial fermentation, strains B1 and C4 could significantly improve the sensory quality of TP. Additionally, the role of B1 and C4 strains on the succession of the microbial community structure after TL and TP fermentation was studied, laying a foundation for a better understanding of the correlation between microbes and tobacco quality.

## Materials and methods

### Tobacco samples and culture conditions

The first-cured tobacco samples KRK26 were collected from Yanshan County, Wenshan Prefecture, Yunnan Province, China in 2020 and used as the test material. TP was crushed and passed through 75 µm screens at low temperature; TL was prepared by removing the tobacco stems.

The culture and isolation of starch, cellulose, and protein-hydrolyzing microorganisms, and the determination of amylase, cellulase, and protease activities were performed under the following conditions: starch medium (10.0 g/L soluble starch, 10.0 g/L tryptone, 5.0 g/L yeast extract, and 5.0 g/L NaCl, pH 7.0), cellulose medium (10.0 g/L carboxymethylcellulose sodium (CMC-Na), 10.0 g/L tryptone, 5.0 g/L yeast extract, 10.0 g/L NaCl, 1.0 g/L K_2_HPO_4_, and 0.5 g/L MgSO_4_•7H_2_O, pH 7.0), and casein medium ( 5.0 g/L casein, 10.0 g/L tryptone, 5.0 g/L yeast extract, and 5.0 g/L NaCl, pH 7.2). The culture conditions of isolated strains were as follows: LB medium (5.0 g/L yeast extract, 10.0 g/L tryptone, and 10.0 g/L NaCl, pH 7.0, sterilized at 115 ℃ for 30 min), potato dextrose agar (PDA) medium (200.0 g/L potato was cut into small pieces, boiled for 20–30 min, and filtered with 8 layers of gauze), and glucose 20.0 g/L, agar 20.0 g/L, natural pH.

### Screening of functional strains from tobacco

After clipping the first-cured tobacco, 10.0 g TL was weighed and inoculated into 250.0 mL flasks containing 90.0 mL sterile water, followed by incubation under 200 rpm shaking at 25 ℃ for 60 min. After gradient dilution (10^–2^-10^–5^), the bacterial culture was plated separately on 10.0% starch, 10.0% CMC-Na, and 5.0% casein agars and incubated at 37 ℃ for 2–3 days. Bacterial colonies with rapid growth and large diameters were picked and purified. Meanwhile, glycerol stocks (50% v/v) were prepared for each colony and stored at – 80 ℃ until further use.

### Identification of isolated strains

The genomic DNA of each isolated strain was extracted by the HiPure Bacterial DNA Kit as instructed by the manufacturer (Magen, China). The extracted DNA was used as template for polymerase chain reactions (PCR) amplification of 16S rDNA using universal primers set: forward primer 27F (5′-AGTTTGATCMTGGCTCAG-3′) and reverse 1492R primer (5′-GCTTACCTTGTTACGACTT-3′). The product of PCR was sequenced in Guangzhou Ruibo Biotechnology Co., Ltd., (Guangdong, China). The sequences determined in this study were assembled using ContigExpress, uploaded to National Center for Biotechnology Information (NCBI) database, and compared with the identified species using the basic local alignment search tool (BLAST). Nucleotide sequences were aligned initially using Clustal W and then adjusted manually. Distance matrices and phylogenetic trees were calculated according to the *p*-distance model (Nei and Kumar 2000) and the neighbor-joining algorithm (Saitou and Nei 1987) using the MEGA 7 (MEGA, USA) software packages (Kumar et al. 2016). One thousand bootstraps were performed to assign confidence levels to the nodes in the trees (Felsenstein 1985).

### Fermentation of different tobacco shapes by microorganisms

TL fermentation: the screened microorganisms B1 and C4 were cultured at the logarithmic growth phase and centrifuged at 8000 rpm for 5 min to collect the cells and resuspended by adding the same volume of sterile water to obtain the bacterial suspension. Finally, 40 mL bacterial suspension was evenly sprayed on the surface of the 100 g TL (KRK26) (Wang et al. 2010; Wei et al. 2014; Wu et al. 2021), put into a sealed food bag, and fermented at 37 °C for 7 days.

TP fermentation: the bacterial suspension was prepared as described above for TL fermentation. Next, TP (100 g, KRK26) was weighed into Tissue Culture Bottles, followed by spraying 40 mL bacterial suspension evenly to the sample under stirring, and fermentation at 37 °C for 7 days (Dai et al. 2020; Zhao et al. 2012).

Meanwhile, control samples were also prepared by treating raw TP/TL samples as described above but without spraying any bacterial suspension.

### Sensory quality assessment

After microbial fermentation, the different treated tobacco samples were directly rolled into “Heat-not-burn” (HnB) cigarettes according to the factory processing standard, followed by evaluation by five experts who have the certificate of qualification for cigarette evaluation and smoking from China Tobacco Yunnan Industrial Co., Ltd. The sensory quality was determined by the new cigarette sensory evaluation method of China Tobacco Yunnan industry-standard QYNZY.J07.022-201 (Additional file 1: Table S1). The result of each total score was the average score of five experts, with quality indexes, including the volume of smoke (10 points), aroma and flavor (30 points), physiological strength (10 points), harmony (10 points), irritancy (15 points), and taste (25 points).

### Determination of aroma components

The content of aroma components in tobacco was determined using the GC/MS fingerprint technique. The different shapes of tobacco samples fermented for 7 days were accurately weighed at 25.0 g, followed by shaking with 20.0 g NaCl and 400.0 mL deionized water in a 1000.0 mL round-bottom flask, treatment in a simultaneous distillation extraction (SDE) device, heating at 60℃ in an electric heating jacket, and finally using NIST98 mass spectrometry library and Agilent chemical workstation for qualitative and quantitative analysis of aroma components, respectively (Li et al. 2020; Zhu et al. 2015).

### Structural characterization of different tobacco shapes

The surface morphology properties of raw TP/TL and fermented TP/TL samples were investigated with a Merlin scanning electron microscope (SEM) (Carl Zeiss, German) operated at a voltage of 10.0 kV, where the samples were put in an aluminum stub with carbon tape and coated with conductive gold (Yan et al. 2020).

X-ray diffraction (XRD) analysis of raw TP/TL and fermented TP/TL samples was performed on an Empyrean (PANalytical B.V, Holland) Diffractometer system at a scan step size of 0.03° with the 2θ values ranging from 5.00° to 50.00°. The crystallinity index (*CrI*) was calculated by the following equation (Yan et al. 2020):

$$CrI = \frac{{I_{002} - I_{am} }}{{I_{002} }} \times 100\backslash \% ,$$

where *I*_*002*_ and *I*_*am*_ represent the intensity of the crystalline peaks at 2θ values of 17.1°/16.9° and 15.7°, respectively (Dome et al. 2020).

Fourier transform infrared spectroscopy (FTIR) (Thermo Nicolet Corporation, USA) was used to measure the spectra of raw TP/TL and fermented TP/TL samples within the wavelength range of 4000–400 cm^−1^ with 32 scans at a spectral resolution of 4 cm^−1^ per sample (Luo et al. 2021).

### Microbial community diversity and structure

To investigate the effect of microbial addition on the microbiota of different tobacco shapes after fermentation, the genomic DNA isolated from different samples was used as a temple for 16S rRNA gene amplification. Bacterial diversity was identified using primers 799F-1193R in the V5-V7 region of 16S rRNA (799F sequence (5′–3′): AACMGGATTAGATACCCKG; 1193R sequence (5′–3′): ACGTCATCCCCACCTTCC). Meanwhile, high-throughput sequencing analysis was used to explore the effects of adding microorganisms on the quality of different shapes of tobacco during fermentation. For principle coordination analysis (PCoA), the significant difference between treatments was tested by using the Adonis. The biomarkers of microbial-fermented tobacco samples were analyzed by Linear Discriminate Analysis (LDA) Effect Size (LEfSe), and taxa with logarithmic LDA higher than 4 were identified as the biomarkers (Jiang et al. 2021; Shen et al. 2018). All the bacterial and fungal raw sequence data were uploaded to the NCBI as a BioProject submission (SUB12245720) with accession number PRJNA897824 (ID: 897824 https://www.ncbi.nlm.nih.gov/bioproject/PRJNA897824).

### Analytical methods

Amylase and cellulase activities were determined by the DNS method of Malhotra (Malhotra et al. 2000) and standard QB/T 2538-2009, respectively. Protease activity was measured using the Chinese standard GB/T 23527-2009.

The contents of total sugar, reducing sugar, starch, total nicotine, cellulose, and protein in tobacco were determined by using the tobacco industrial standard methods (YC/T 159-2019, YC/T 216-2013, YC/T 468-2013, YC/T 347-2010, and YC/T 249-2008), respectively.

### Statistical analysis

The results are presented as means ± standard deviation of three replicates of independent experiments. Inoculants (*Bacillus subtilis* B1 and *Cytobacillus oceanisediminis* C4) and different tobacco shapes (TP and TL) were dependent variables. The fermentation time of 7 d, fermentation temperature of 37 ℃, and 40% inoculum amount were fixed factors in statistical analysis. Statistical analysis was performed using the software SPSS 26.0 (IBM, USA). ANOVA and Duncan’s multiple range tests were applied to determine significant differences. A value of *p* < 0.05 was considered a statistically significant difference.

## Results

### Isolation and identification of strains

#### Enzyme activity analysis of strains

**Fig. 1:**
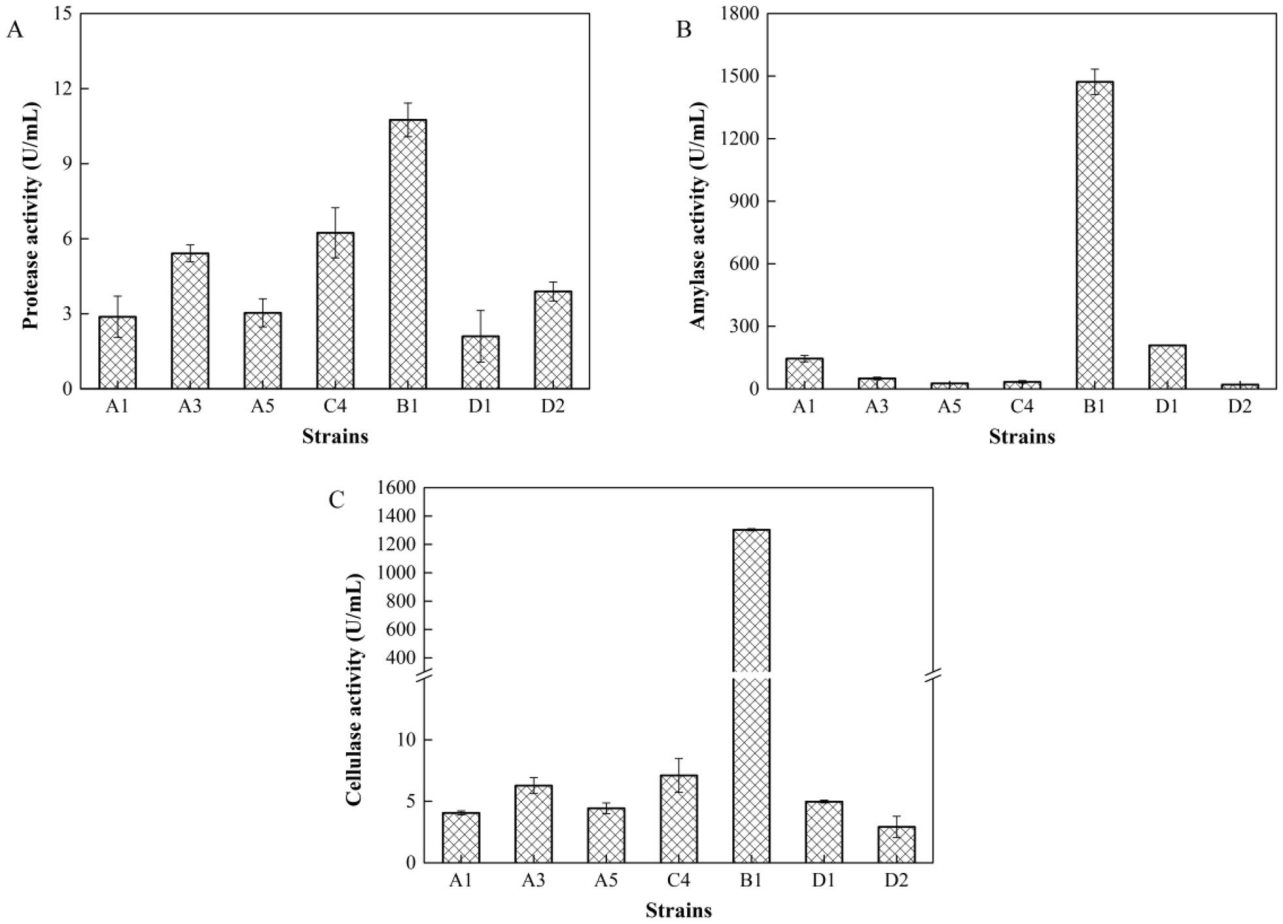
Activity of enzymes in tobacco strains. **A** Protease; **B** Amylase; **C** Cellulase

Seven strains were obtained from tobacco through different-sized hydrolytic zones of different selective cultures. D1 and D2 strains were fungi, and the remaining five strains (A1, A3, A5, C4, and B1) were bacteria. The amylase, cellulase, and protease activities of fungi and bacteria were measured after 24 h and 12 h of fermentation, respectively (Fig. 1). The results indicated that strain B1 was significantly higher than the other strains in the measured amylase activity (Fig. 1B, 1472.159 U/mL) and cellulase activity (Fig. 1C, 1303.030 U/mL). Meanwhile, strains B1 and C4 had higher protease activity (10.749 U/mL and 6.237 U/mL, respectively) than the other strains (Fig. 1A), and strain C4 had the amylase and cellulase activities of 33.562 U/mL and 7.107 U/mL, respectively.

#### Identification of Strains

**Fig. 2:**
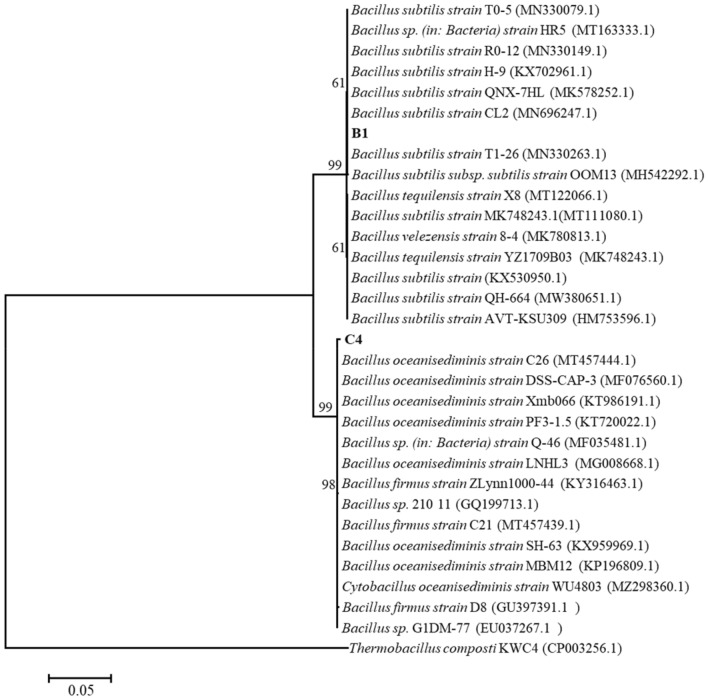
Neighbor-joining tree of tobacco strains B1 and C4 and related bacterial strains based on a neighbor-joining algorithm of the 16S rDNA sequence. Bootstrap values are shown as the percentage of 1000 replicates. The bar (0.05) at the bottom of the tree indicates the substitution per nucleotide position

Because of their high amylase, cellulase, and protease activities, a phylogenetic tree of strains B1 and C4 was constructed based on 16S rDNA gene sequences using MEGA7.0 software. BLAST analysis of the 16S rDNA gene sequences suggested that strains B1 and C4 were close to the genus *Bacillus sp.* and *Cytobacillus sp.*, respectively (Fig. 2), with the highest sequence identity (99.72% and 99.37%, respectively.) with *Bacillus subtilis* strain T1-26 (GenBank accession number MN330263.1) and *Bacillus oceanisediminis* strain C26 (GenBank accession number MT457444.1), respectively. According to the detection results of Bergey’s Manual of Determinative Bacteriology (Bergey 1994) and Handbook for Identification of Common bacterial systems, the bacterial strains B1 and C4 were identified as *Bacillus subtilis* and *Cytobacillus oceanisediminis*, respectively (Additional file 1: Table S2). The nucleotide sequences for strains B1 and C4 were submitted to GenBank with accession numbers of ON428436 (https://www.ncbi.nlm.nih.gov/nuccore/ON428436) and ON428437 (https://www.ncbi.nlm.nih.gov/nuccore/ ON428437), respectively.

### Fermentation of tobacco by microorganisms

#### Effects of microorganism fermentation on the chemical composition of tobacco

**Fig. 3:**
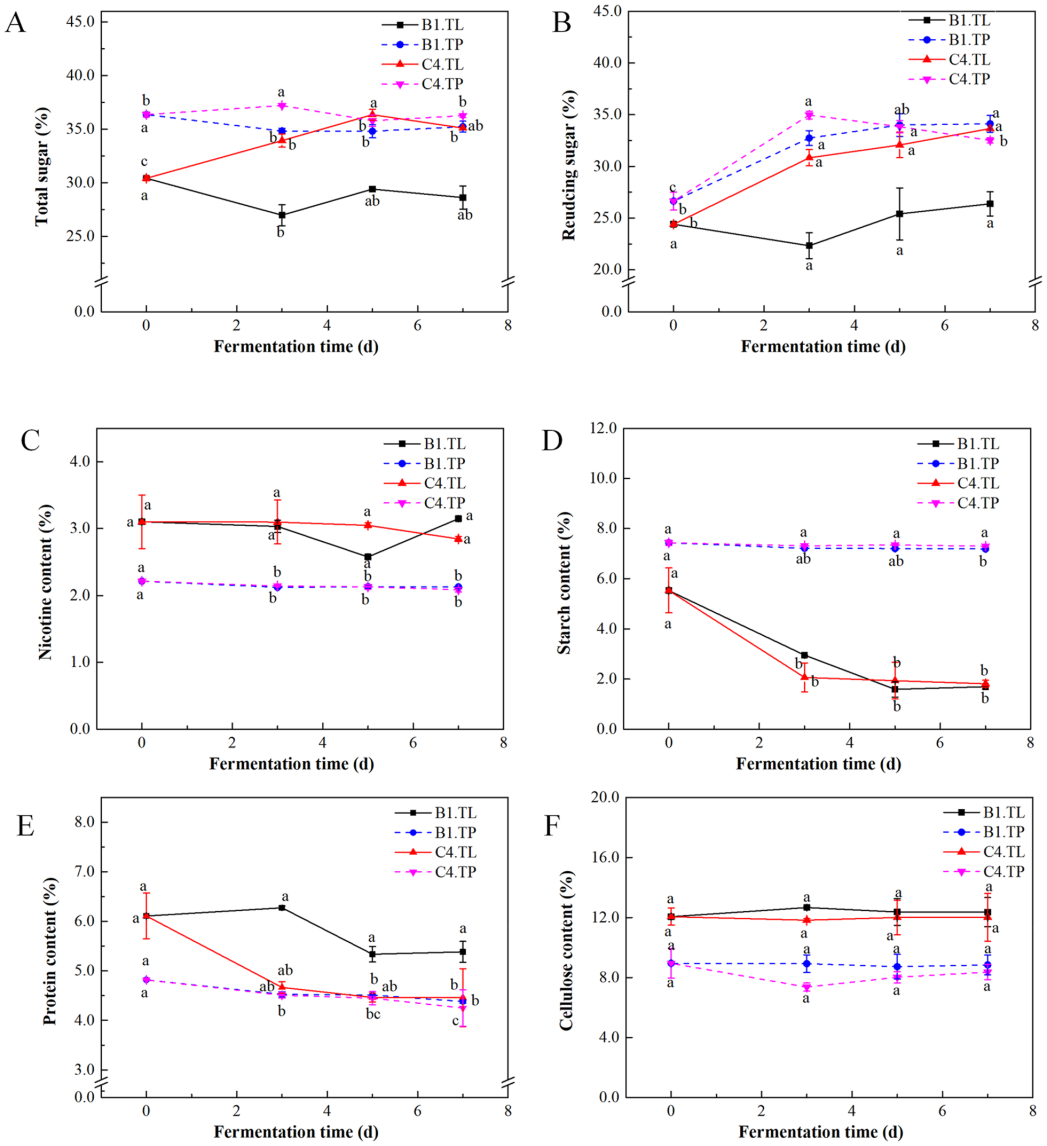
Effects of microbial fermentation on chemical compositions in tobacco powder (TP) and tobacco leaves (TL) groups. **A** Total sugar; **B** Reducing sugar; **C** Nicotine; **D** Starch; **E** Protein; **F** Cellulose. Different small letters (a, b) indicate significant difference at *p* values < 0.05, and the same letter indicates no significant difference

The total sugar (Fig. 3A) of TP and TL had a varying degree of changes during strains C4 and B1 fermentation. Compared with their respective controls, C4.TP, C4.TL, and B1.TP showed an increase of 22.06%, 37.92%, and 28.08% in reducing sugar content (Fig. 3B) after fermentation, in contrast to only 8.12% for B1.TL. The nicotine content (Fig. 3C) was significantly reduced (*p* < 0.05) in C4.TP and B1.TP, and showed no significant change in C4.TL and B1.TL compared to the corresponding control, respectively. The starch content (Fig. 3D) was decreased significantly (*p* < 0.05) in C4.TL, B1.TL, and B1.TP, but was not significantly different in C4.TP relative to the corresponding control, respectively. The protein content (Fig. 3E) decreased by 11.83% and 9.10% in C4.TP and B1.TP relative to TP control, and from 6.11% to 4.38% and 5.39% in C4.TL and B1.TL, respectively. The cellulose content (Fig. 3F) showed no significant change in all the groups after fermentation.

#### Effects of fermentation on tobacco aroma components

The content of aroma components of TP and TL samples are shown in Table 1 and Table 2, respectively. The results showed that neophytadiene, solanone, aromadendrene, dibutyl phthalate, β-damascone, alloaromadendrene, megastigmatrienone B, C, and D, and dihydroactinidiolide were all increased in TP and TL samples fermented by both C4 and B1 strains. These components played an important role in increasing tobacco fragrances, enhancing tobacco smoking comfort, and improving overall smoking sense. Compared with their corresponding control, the total aroma components increase rate was in the order of B1.TL (146.53%) > C4.TP (128.73%) > C4.TL (107.94%) > B1.TP (102.71%). Meanwhile, we found that neophytadiene with the highest proportion of aroma substances in tobacco, and except for neophytadiene, the total content of the other aroma components was in the order of C4.TP (680.12 μg/g) > B1.TP (649.65 μg/g) > B1.TL (593.59 μg/g) > C4.TL (486.28 μg/g).

**Table 1:** The content of aroma components in fermented tobacco powder (TP) with different microbial fermentations

Components (μg/g)	Control.TP	C4.TP	B1.TP	C4.TP increase rate (%)	B1.TP increase rate (%)
Neophytadiene	316.78	567.63	456.17	+ 79.19	+ 44.00
Black-pinitol	12.13	95.15	2.97	+ 684.20	− 75.55
Solanone	23.20	64.95	59.29	+ 180.01	+ 155.63
Aromadendrene	2.55	56.24	39.06	+ 2104.23	+ 1430.78
Phytol	9.87	51.07	22.21	+ 417.31	+ 124.99
Dibutyl phthalate	11.76	42.27	40.40	+ 259.33	+ 243.38
Methyl palmitate	7.55	39.82	7.36	+ 427.76	− 2.49
β-Damascone	3.09	30.32	27.26	+ 882.21	+ 783.10
Alloaromadendrene	4.10	25.38	66.50	+ 518.69	+ 1520.69
Megastigmatrienone B	6.47	23.52	24.31	+ 263.38	+ 275.53
Dihydroactinidiolide	0.75	17.35	15.99	+ 2214.94	+ 2032.55
Cembrenediol 3	4.01	15.37	18.76	+ 282.82	+ 367.38
Geranyl acetone	7.15	14.91	14.72	+ 108.53	+ 105.74
Megastigmatrienone D	5.67	12.66	4.73	+ 123.15	− 16.70
Artemisia triene	2.83	10.89	18.79	+ 284.69	+ 563.83
Others	98.08	121.21	179.57	+ 41.25	+ 125.21
Total aroma (except neophytadiene)	228.73	680.12	649.65	+ 197.35	+ 184.02
Total aroma	545.51	1247.75	1105.82	+ 128.73	+ 102.71

The Control.TP group represents unfermented tobacco powder. The C4.TP and B1.TP represent tobacco powder (TP) fermented for 7 days by microorganisms C4 and B1, respectively

**Table 2:** The content of aroma components in fermented tobacco leaves (TL) with different microbial fermentations

Components (μg/g)	Control.TL	C4.TL	B1.TL	C4.TL increase rate (%)	B1.TL increase rate (%)
Neophytadiene	586.23	979.67	1144.25	+ 67.11	+ 95.19
Black-pinitol	4.09	5.11	8.30	+ 24.72	+ 102.82
Solanone	0.34	27.67	46.91	+ 7946.61	+ 13540.38
Aromadendrene	3.52	16.24	22.25	+ 361.42	+ 532.32
Phytol	3.81	0.34	7.06	− 91.08	+ 85.15
Dibutyl phthalate	1.62	38.55	34.13	+ 2273.24	+ 2001.20
Methyl palmitate	4.20	2.76	36.02	− 34.31	+ 756.57
β-Damascone	10.68	29.52	38.07	+ 176.32	+ 256.41
Alloaromadendrene	0.74	19.54	30.30	+ 2535.52	+ 3986.06
Megastigmatrienone B	6.53	38.23	50.35	+ 485.44	+ 671.05
Dihydroactinidiolide	2.03	10.56	9.79	+ 420.03	+ 382.05
Cembrenediol 3	2.06	4.82	7.41	+ 134.01	+ 259.74
Geranyl acetone	2.47	5.55	3.91	+ 124.31	+ 58.28
Megastigmatrienone D	5.87	35.11	41.09	+ 497.69	+ 599.53
Artemisia triene	1.66	3.58	2.76	+ 116.41	+ 66.88
Others	69.12	248.70	255.22	+ 259.80	+ 269.23
Total aroma (except neophytadiene)	118.77	486.28	593.59	+ 309.42	+ 399.78
Total aroma	705.00	1465.95	1737.84	+ 107.94	+ 146.53

The Control.TL group represents unfermented tobacco leaves. The C4.TL and B1.TL represent tobacco leaves (TL) fermented for 7 days by microorganisms C4 and B1, respectively

#### Effects of microorganism fermentation on tobacco sensory quality

The effects of B1 and C4 on the sensory quality of different tobacco shapes were investigated and the results are shown in Table 3. Compared with their respective controls, the microbial-fermented samples showed a varying degree of improvement in sensory quality, with the most obvious improvement for C4.TP (total score 84.00), where the indicators of aroma and flavor and harmony have been significantly improved (*p* < 0.05) relative to TP control, respectively. Meanwhile, C4.TP was characterized by unique style, great coordination, rich and elegant aroma, suitable strength, good oral aroma, excellent suction feeling, etc. For B1-fermented samples, B1.TP had a total score of 83.50, where indicators of aroma and flavor were significant improvement (*p* < 0.05) relative to TP control. Moreover, B1.TL showed a similar trend to C4.TL in sensory quality evaluation, with their physical strength indicator superior to that of Control.TL, and aroma and flavor weaker than that of Control.TL. Collectively, according to the sensory descriptions of cigarette smoking by five experts, TP showed better performance than TL in overall texture and skeleton of all samples, coupled with smooth smoke and good permeability, with the best overall effect on TP sensory quality by microbial C4 (Additional file 1: Table S3).

**Table 3:** Effect of microbial fermentation on the sensory quality of cigarettes in tobacco powder (TP) groups and tobacco leaves (TL) groups

Groups	Volume of smoke (10–0)	Aroma and flavor (30–0)	Physiological strength (10–0)	Harmony (10–0)	Irritancy (15–0)	Taste (25–0)	Total (100–0)
Control.TP	8.00 ± 0.00^*a*^	22.83 ± 0.24^*b*^	8.00 ± 0.00^*a*^	8.00 ± 0.00^*b*^	13.00 ± 0.00^*a*^	22.17 ± 0.24^*a*^	82.00 ± 0.41^*b*^
C4.TP	8.17 ± 0.24^*a*^	23.50 ± 0.00^*a*^	8.33 ± 0.24^*a*^	8.33 ± 0.24^*a*^	13.17 ± 0.24^*a*^	22.50 ± 0.00^*a*^	84.00 ± 0.41^*a*^
B1.TP	8.17 ± 0.24^*a*^	23.33 ± 0.24^*a*^	8.33 ± 0.24^*a*^	8.00 ± 0.00^*b*^	13.33 ± 0.24^*a*^	22.33 ± 0.24^*a*^	83.50 ± 0.41^*a*^
Control.TL	8.17 ± 0.24^*a*^	23.17 ± 0.24^*a*^	7.83 ± 0.24^*a*^	7.83 ± 0.24^*a*^	13.17 ± 0.24^*a*^	22.83 ± 0.24^*a*^	83.00 ± 0.41^*a*^
C4.TL	8.17 ± 0.24^*a*^	23.17 ± 0.24^*a*^	8.17 ± 0.24^*a*^	8.17 ± 0.24^*a*^	13.17 ± 0.24^*a*^	22.67 ± 0.24^*a*^	83.50 ± 0.41^*a*^
B1.TL	8.33 ± 0.24^*a*^	23.00 ± 0.00^*a*^	8.17 ± 0.24^*a*^	8.17 ± 0.24^*a*^	13.17 ± 0.24^*a*^	22.67 ± 0.24^*a*^	83.50 ± 0.41^*a*^

Values are means ± standard deviations (*n* = 3). Different letters (a, b) show statistically significant differences within each tobacco powder/tobacco leaves group (*p* < 0.05), and the same letter indicates no significant difference

### Structural characterization

#### The SEM analysis

**Fig. 4:**
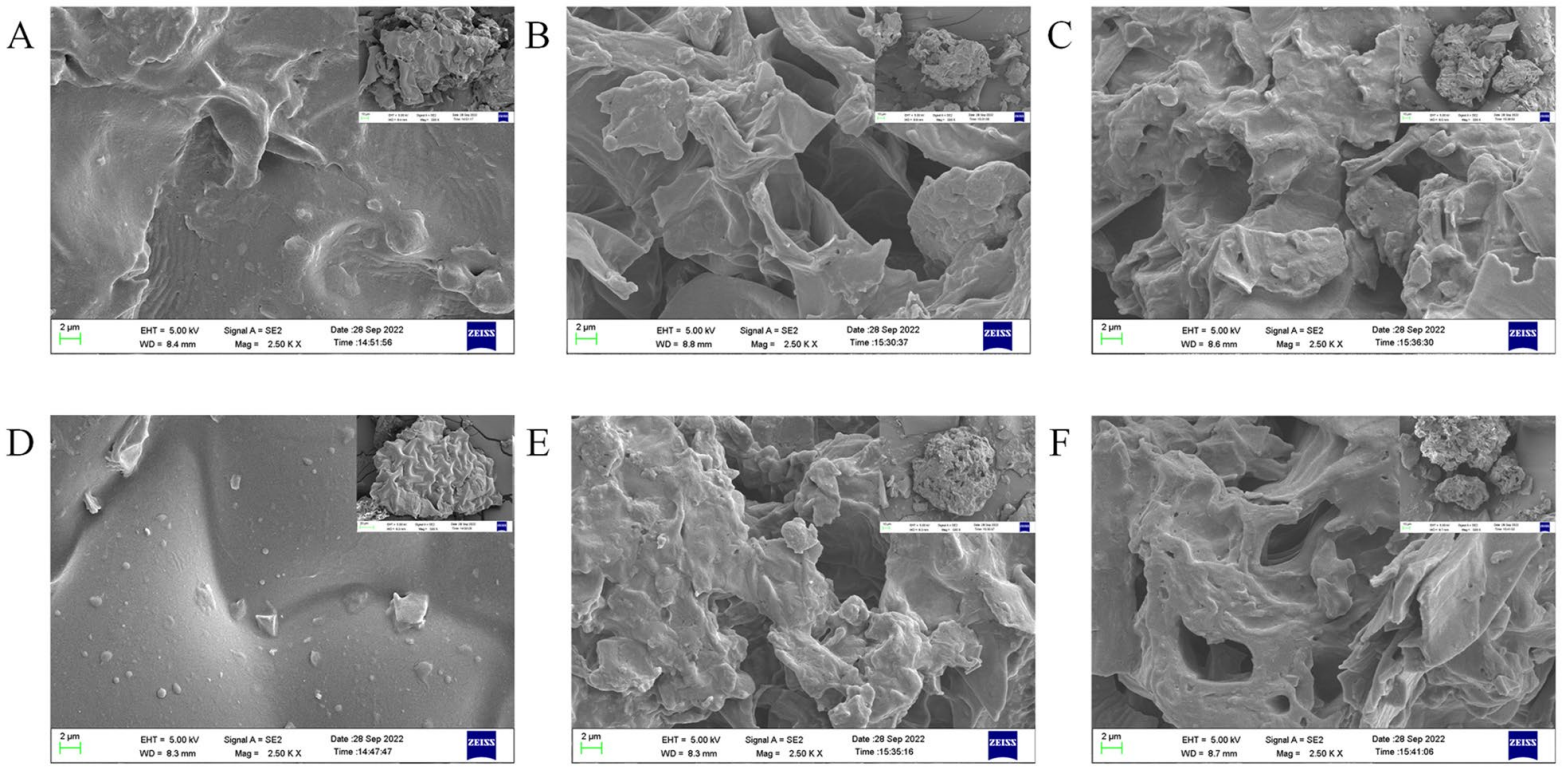
SEM images of microbial-fermented tobacco powder (TP) and tobacco leaves (TL). **A** Control.TP; **B** C4.TP; **C** B1.TP; **D** Control.TL; **E** C4.TL; **F** B1.TL

The SEM images of raw, microbial C4 and B1-fermented TP and TL samples were observed (Fig. 4). The surface morphological characteristics of Control.TP (Fig. 4A) and Control.TL (Fig. 4D) were seen to have a complete structure, with a closely connected and smooth surface, in contrast to a more loose, rough, wrinkled, and porous surface after microbial C4 (Fig. 4B, E) and B1 (Fig. 4C, F) fermentation. After C4 fermentation, the surface structure showed more folds in TP (Fig. 4B) than in TL samples (Fig. 4E). The results showed that microbial fermentation could change the surface structure of TP/TL.

#### The FTIR spectroscopic analysis

The FTIR spectra of TP and TL (Additional file 1: Fig. S1A, B) fermented by different microbes indicated that the absorption band was around 3302 cm^−1^ (Zhao et al. 2013) and 1377 cm^−1^ (Zhao et al. 2015a), corresponding to O–H bonds and symmetric in-plane bending vibration of -CH_3_ for diverse substances, such as cellulose, respectively. The absorption bands at around 2924 cm^−1^ (He et al. 2019) were attributed to the characteristic vibrations of C-H from polysaccharides and proteins. The bands near 1741 cm^−1^ (Zhao et al. 2015b) and 1602 cm^−1^ (Ramachandraiah and Chin 2016) were related to C≡O stretching bands and aromatic C-H bonds, respectively. The absorption at around 1030 cm^−1^ (Zhao et al. 2015b) represented the stretching vibration of C-O group, corresponding to the original pyran ring of the polysaccharide. Meanwhile, similar chemical spectra were observed in different tobacco shapes under microbial fermentation, indicating similar types of chemical constitution in TP and TL (Zhang et al. 2022). The above results showed that no new group bands were generated under different microbial fermentations.

#### The XRD analysis

XRD analysis was performed for the crystallinity of Control.TP, C4.TP, B1.TP, Control.TL, C4.TL, and B1.TL (Additional file 1: Fig. S2A, B). In the XRD spectrum, TP and TL showed a strong diffraction peak of around 16.9°/17.1° and a weak diffraction peak around 15.7°, respectively, similar to the crystal structure of B-type crystalline starch (Dome et al. 2020; Sun et al. 2015). The XRD peak intensity and area were wider in TP samples than in TL samples, probably due to the increased contribution of amorphous tobacco. The raw and microbial-fermented tobacco samples showed no significant change in the characteristic peaks of XRD patterns, indicating that the tobacco crystalline feature was not altered by the microbial fermentation.

### Microbial community diversity and structure

#### Microbial community diversity

**Fig. 5:**
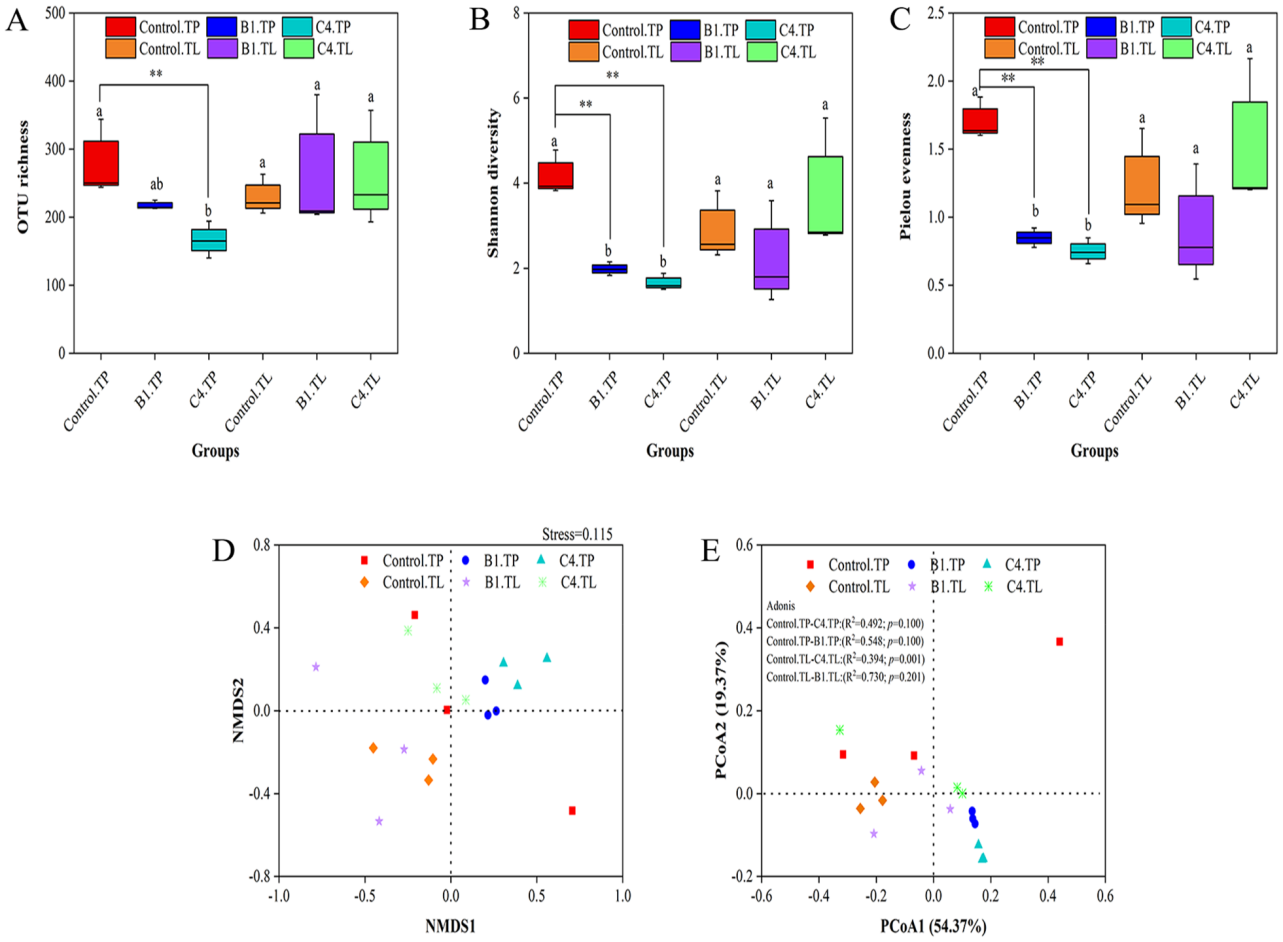
Analysis of microbial α-diversity and β-diversity in fermented tobacco powder (TP) and tobacco leaves (TL). **A** OTU richness; **B** Shannon diversity; **C** Pielou evenness; **D** NMDS; **E** PCoA. Different letters (a, b) represent statistically significant differences within each tobacco powder/tobacco leaves group (*p* < 0.05), * represents statistical significance in TP/TL (*p* < 0.05), ** represents statistical significance in TP/TL (*p* < 0.01)

After termination of fermentation, samples were collected from the Control.TP, C4.TP, B1.TP, Control.TL, C4.TL, and B1.TL for microbial community analysis (Fig. 5). The bacterial microbial α-diversity of inoculated microorganisms on different tobacco morphological samples was further evaluated in terms of OUT richness (Fig. 5A), Shannon diversity (Fig. 5B), and Pielou evenness (Fig. 5C). The coverage of all samples was more than 99%, and microbial fermentation was found to have a great effect on the α-diversity of the TP microbial community, with a significant decrease in OUT richness, Shannon diversity, and Pielou evenness. However, TL samples had no significant changes in microbial community α-diversity after microbial fermentation. The results showed that the microbial richness, diversity, and uniformity changed after inoculation of strains C4 and B1 into TP and fermentation.

We further evaluated the effect of microorganisms on the microbial community structure of different tobacco morphologies by PCoA (Fig. 5E) and non-metric multidimensional scaling (NMDS) analysis (Fig. 5D). In NMDS analysis, a stress value < 0.2 is generally considered meaningful, and the stress value of 0.11 indicated that the virtual axis had a good explanatory meaning for bacterial community (NMDS) analysis. The PCoA analysis showed that the first two axes could explain 73.74% of bacterial community variation. According to PCoA, there were significant differences between Control.TP-Control.TL (Adonis; R^2^ = 0.560, *p* = 0.001) and Control.TL-C4.TL (Adonis; R^2^ = 0.394, *p* = 0.001), while no significant differences between the other groups. The results showed that TP and TL were different in microbial diversity.

#### Taxonomic composition

**Fig. 6:**
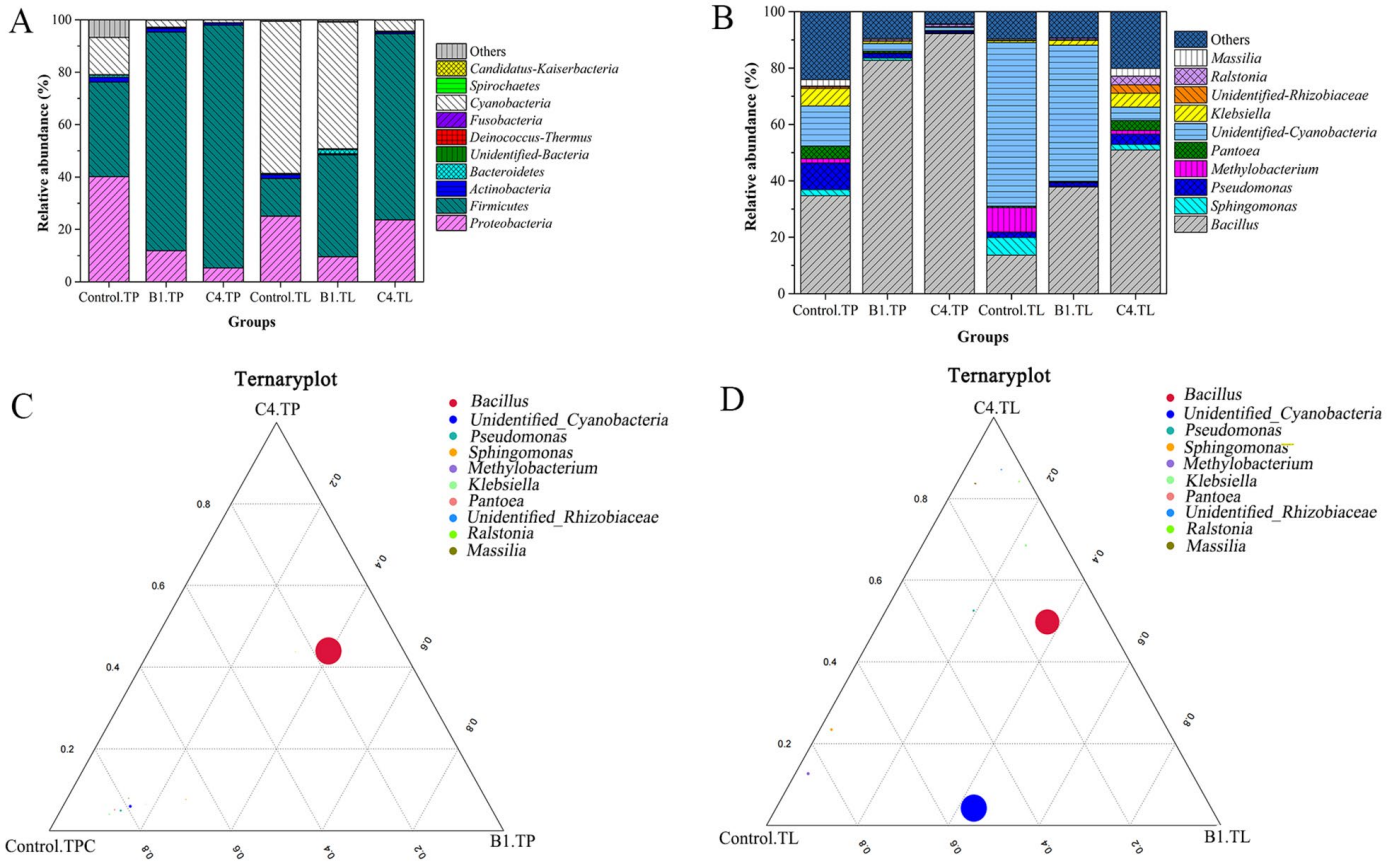
Difference between tobacco powder (TP) and tobacco leaves (TL) groups in microbial community after tobacco fermentation. **A** Phyla level; **B** Genus level; **C** The ternary plot of TP group was at genus level; **D** The ternary plot of TL group at genus level. Each circle represents one genus. The size of each circle represents its relative abundance (weighted average). The position of each circle was determined by the contribution of the indicated compartments to the total relative abundance

The effects of microbial fermentation on the microbial community structure of different tobacco morphologies were further investigated by analyzing the microbial distribution of different groups at phylum (Fig. 6A) and genus levels (Fig. 6B). The results showed that by adding microorganisms for 7 days of fermentation, B1.TP and C4.TP showed phylum dominance of *Firmicutes* (83.41% and 92.68%), *Proteobacteria* (11.89% and 5.27%), and *Cyanobacteria* (2.66% and 1.07%), in contrast to the main phyla of *Firmicutes* (38.89% and 51.63%), *Proteobacteria* (9.56% and 23.65%), and *Cyanobacteria* (48.33% and 4.70%) for B1.TL and C4.TL. Compared to Control.TP and Control.TL, adding microbes significantly increased the relative abundance of *Firmicutes*, while decreasing the relative abundance of *Proteobacteria* and *Cyanobacteria.* At the genus level (Fig. 6B), *Bacillus* (82.70% and 92.14%), *Sphingomonas* (0.86% and 0.25%), and *Unidentified-Cyanobacteria* (2.66% and 1.07%) were the dominant bacterial B1.TP and C4.TP groups, while *Bacillus* (37.81% and 50.96%), *Sphingomonas* (0.22% and 1.98%), and *Unidentified-Cyanobacteria* (48.29% and 4.69%) were the primary genera in B1.TL and C4.TL groups. The ternary plot showed that *Bacillus* was biased to C4.TP and B1.TP, with the highest *Bacillus* abundance in TP group (Fig. 6C). In TL group, *Bacillus* was biased to C4.TL and B1.TL, and *Unidentified-Cyanobacteria* was inclined to Control.TL and B1.TL (Fig. 6D). These results were consistent with the results of genus-level abundance (Fig. 6B). Addition of microorganisms significantly increased the relative abundance of *Bacillus* in TP and TL, indicating that *Bacillus* was highly enriched. Based on the bacterial community heat map at the genus level (top 35), TP and TL samples had different bacterial communities at taxonomic classification levels (Additional file 1: Fig. S3).

**Fig. 7:**
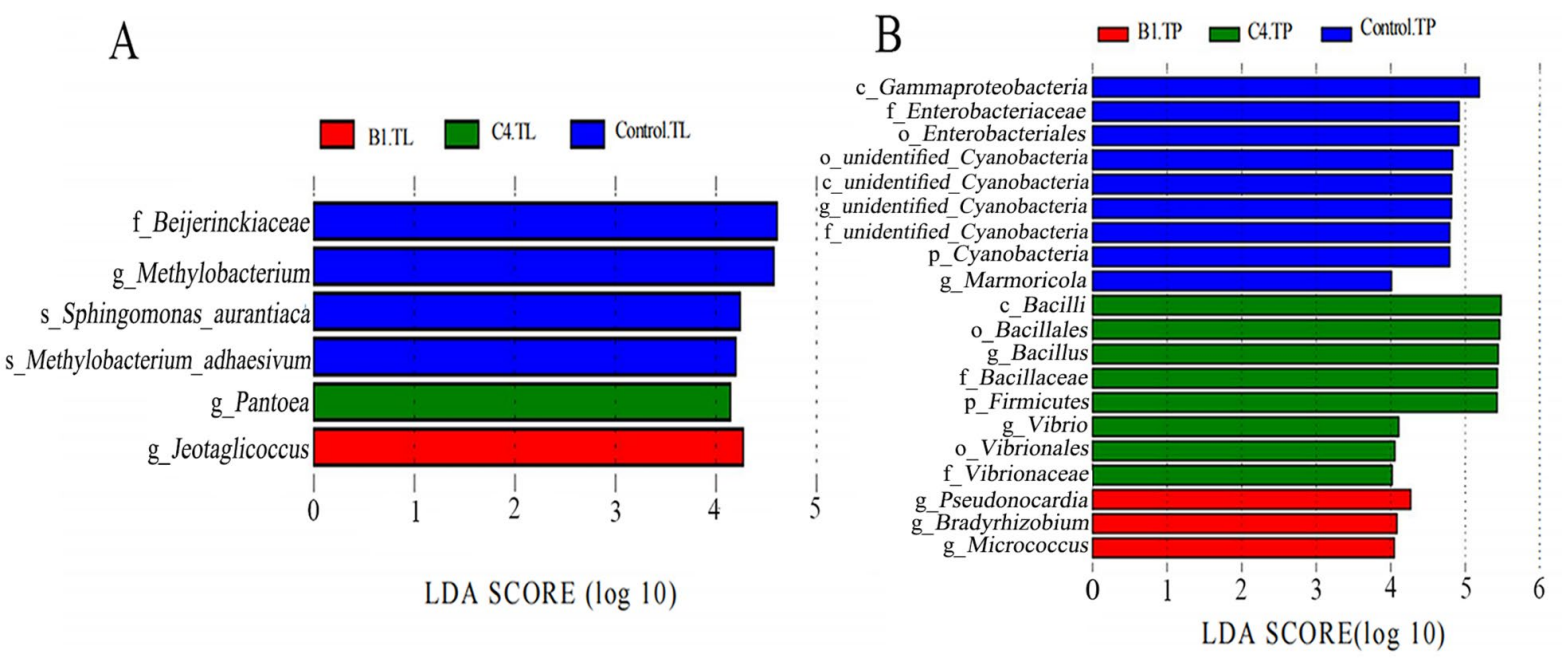
LEfSe analysis of the taxonomic biomarker in the bacterial community of control and fermented tobacco leaves (TL) and tobacco powder (TP). **A** TL group; **B** TP group

The LEfSe results showed that the content of taxonomic biomarkers was higher in TP group than in TL group. In the TP group (Fig. 7B and Additional file 1: Fig. S4A), *Bacilli* and *Pseudonocardia* were important biomarkers in C4.TP and B1.TP, respectively, and *Gammaproteobacteria* and *Enterobacteriaceae* were the biomarkers in Control.TP. In TL group (Fig. 7A and Additional file 1: Fig. S4B), *Pantoea* and *Jeotgalicoccus* were the main biomarkers in C4.TL and B1.TL, respectively, and *Beijerinckiaceae* and *Methylobacterium* were the biomarkers in Control.TL. However, no *Bacilli* biomarker was identified in B1.TP, C4.TL, and B1.TL groups.

## Discussion

In order to alleviate the adverse effects of starch, protein, and cellulose in first-cured tobacco leaves on the sensory quality of tobacco products, functional microorganisms were screened from the microbial flora of tobacco raw materials. We identified *Cytobacillus oceanisediminis* C4 and *Bacillus subtilis* B1, two *Bacillus. sp*. with high amylase, protease, and cellulase activities (Fig. 1 and Fig. 2). According to previous reports, *Bacillus sp.* was the dominant bacterium in tobacco (Wang et al. 2018a), which could effectively degrade starch, protein, and cellulose in tobacco (Dai et al. 2020). Among them, *Bacillus subtilis* mainly isolated from tobacco root, soil, and surface, thus playing a role in accelerating the ideal taste formation of cigars (English et al. 1967) and controlling tobacco black shank (Han et al. 2016). *Cytobacillus oceanisediminis* C4 was the first strain isolated from tobacco, which was mainly obtained from marine sediments (Zhang et al. 2010) and rice roots (Bano et al. 2022) in previous studies. Some studies have reported that *Bacillus oceanisediminis* 2691 has a variety of heavy metal-sensing regulators and efflux pumps (Jung et al. 2016) and *Bacillus oceanisediminis* CCMMB584 can control tomato gray mold disease (Berrada et al. 2012), but no further related information is available. The chemical composition of different tobacco shapes varies during storage, but few studies have been performed on the fermentation of different shapes of tobacco (Pereira et al. 2016; Qin and Gong 2016). In this study, CMC was used as the substrate for cellulase determination according to Dai et al. (Dai et al. 2020). CMC, which is different from crystalline cellulose, is generally used as the substrate of β-Glucosidase, indicating the difference between the measured cellulase activity and the actual cellulase activity. In this study, we investigated the effects of *Cytobacillus oceanisediminis* C4 and *Bacillus subtilis* B1 on the quality of different shapes of tobacco (TP and TL).

Our results indicated that microorganisms could improve the sensory quality of TP and TL, with a total score of 84.0 for C4.TP, higher than that of the other treatments (Table 3). The microbes could regulate the chemical composition of TP and TL, such as total sugar (Fig. 3A), reducing sugar (Fig. 3B), nicotine (Fig. 3C), starch (Fig. 3D), protein (Fig. 3E), and cellulose (Fig. 3F). Several studies have shown that reducing sugars have a positive effect on the smoking characteristics of tobacco, such as improving flavor and aroma (Li et al. 2003). The content of reducing sugars was significantly higher in C4/B1.TP than in the corresponding control, respectively (Fig. 3A). Meanwhile, the microorganisms could promote the degradation of starch (Fig. 3D) and protein (Fig. 3E) in TP and TL, favoring the formation of aromatic compounds. The accumulation of reducing sugars and amino acids could promote the formation of precursor products for Maillard reaction. There were many kinds of Maillard reaction products with complex structure, including aldehydes, N-substituted glycosylamines, nitrogenous heterocyclic compounds, and Amadori compounds. The pyrolysis products of Amadori compound were mainly aldehydes, pyrazines, and pyrrole, etc., which were important fragrant substances for cigarette. Pyrazines and pyrrole could enhance the fullness of cigarette fragrance. The total amount of precursor products of Maillard reaction of TP and TL increased after microbial fermentation (Additional file 1: Tables S4 and S5), thereby endowing tobacco with a unique sweet and caramel flavor, enhancing the aroma and flavor of tobacco, and increasing the elegance and aroma richness of tobacco products, which were consistent with literature reports (Banožić et al. 2020; Mitsui et al. 2015; Song et al. 2009). In this study, the nicotine content in TP was significantly reduced, and protein content in TP and TL was also decreased by microbial B1 and C4 fermentation. Excessive nicotine in tobacco would be harmful to human body (Liu et al. 2015), and protein could produce the smell of charred feather and harmful substances, such as quinoline, cyanic acid, and benzopyrene with cigarettes smoking (Wu et al. 2021). The results proved that microorganism B1 and C4 could regulate the chemical composition of tobacco and degrade harmful substances of tobacco in the fermentation process, thus reducing the harmful substances in cigarettes.

On the other hand, strains C4 and B1 could promote the accumulation of total aroma components in TP and TL, respectively (Tables 1 and 2). Strains B1 and C4 could accelerate the degradation of starch, protein, and other substances to form aroma compounds, and increase the content of aroma components in tobacco during tobacco fermentation. Among them, neophytadiene had the highest proportion in tobacco, which was mainly derived from the degradation and transformation of chlorophyll in plants (Palic et al. 2002). Neophytadiene could carry tobacco aroma substances into the smoke during the burning process of tobacco, could reduce the irritation of tobacco, increase the alcohol and aroma substances, and could be used as an aroma enhancer for tobacco (Mitsui et al. 2015). Meanwhile, total aroma content and their respective odor threshold values were also important factors affecting tobacco quality (Wang et al. 2018b; Yang et al. 2016). Some researchers have reported that the odor threshold value was low for solanone and β-damascone, but higher for neophytadiene (Alagić et al. 2002; Palic et al. 2002). In the present study, the contents of solanone and β-damascone were higher in TP than in TL (Tables 1 and 2), the proportion of neophytadiene was high, its odor threshold values were high, and its contribution to cigarettes aroma may be smaller than that other aroma components (such as solanone and β-damascone) with high aroma contents and low odor threshold values, which was one of the reasons for the higher overall suction quality of TP cigarettes than TL cigarettes. β-Damascone was of fruit aroma, which could increase tobacco aroma (Ding et al. 2013). According to previous reports, solanone had the fragrance of fresh carrots (Johnson and Nicholson 1965), which could increase the tobacco aroma and make smoke full, mellow and delicate. β-Damascone and solanone played important roles in the contribution of cigarette aroma. Black pinitol could be used as spices and enhance the aroma of tobacco. Aromadendrene was a rare sesquiterpene, which could be used as a spice, endowing elegant fragrance of cigarette. The neophytadiene, megastigmatrienone B, C, and D could increase the alcohol and comfort of tobacco smoke; dihydroactinidiolide and geranylacetone had fruit aroma, which could enhance tobacco scent (Banožić et al. 2020). The accumulation of aroma components played an important role in increasing the fragrance of tobacco and improving the smoking quality of cigarettes. Meanwhile, microbial fermentation of *Cytobacillus oceanisediminis* C4 and *Bacillus subtilis* B1 made TP and TL decomposed by some active enzymes of microorganisms, and enabled the surface structure to be looser in TP and TL than in the corresponding control (Fig. 4), which was conducive to the accumulation of tobacco aroma substances (Yuan et al. 2019).

*Bacillus* was highly enriched after fermentation, accounting for 92.14% in C4.TP (Fig. 6B), higher than that of the other groups (Huang et al. 2010; Wang et al. 2018a; Zhang et al. 2020). LEfSe analysis showed that the potential key biomarkers observed from TP and TL samples might be beneficial to improve tobacco quality (Fig. 7, Additional file 1: Fig. S4). A large number of studies have shown that *Bacillus* plays an important role in regulating chemical composition, promoting accumulation of aroma compounds, and improving tobacco quality (Dai et al. 2020; Wei et al. 2014; Wu et al. 2021). *Pseudonocardia* was a beneficial microbe in healthy tobacco soil, which could promote plant health and xylan degradation (Wang et al. 2017). *Pantoea* was the dominant microorganism for aging flue-cured tobacco surface (Su et al. 2011), which might cause high levels of lipopolysaccharide in cigarettes and tobacco smoke (Chopyk et al. 2017). *Jeotgalicoccus* could rapidly metabolize reducing sugars and organic acids in the early stages of tobacco fermentation to affect tobacco aroma (Di Giacomo et al. 2007). The microorganisms had more influence on the diversity of TP than that of TL microbial community, probably due to the more balanced adaption of species in TP microbial community to fermentation environment. Because strains B1 and C4 belong to different microorganisms, the changes of aroma components and chemical components were different. And the relative abundance of *Bacillus* in C4.TP was higher than that in B1.TP, and the types of microbial biomarkers in C4.TP were higher than those in B1.TP, which may be one of the reasons for the better sensory evaluation quality of C4.TP than B1.TP. Meanwhile, adding microbial C4 and B1 separately could form a synergistic effect with other microorganism taxa to promote the improvement of tobacco quality.

## Conclusions

In conclusion, *Cytobacillus oceanisediminis* C4 and *Bacillus subtilis* B1 isolated from tobacco could play a key role in the fermentation process of different tobacco morphologies (TP and TL) by regulating chemical composition, improving aroma components, and changing tobacco surface structure and tobacco microbiota. *Cytobacillus oceanisediminis* C4 and *Bacillus subtilis* B1 could promote the degradation of nicotine, starch, and protein in TP and TL, leading to increasing reducing sugars. The combination of strain C4 and TP was more favorable to the accumulation of aroma components, such as neophytadiene, solanone, aromadendrene, dibutyl phthalate, and β-damascone. Moreover, the tobacco sensory quality was significantly improved with a total score of 84.0 for C4.TP, which was much higher than that of others. Meanwhile, the addition of strains C4 and B1 could change the structure of microbial communities in tobacco, and *Bacillus* was highly enriched during fermentation, accounting for 92.14% in C4.TP, which indicated that *Bacillus* plays a crucial role in improving tobacco quality. The present work suggests that fermentation of tobacco with exogenous inoculation in TP is feasible and lays a foundation for a better understanding of the correlation between microbes and tobacco quality.

### Supplementary Information

**Additional file 1: Figure S1**. FTIR spectra of raw and various microbial fermentation different tobacco shapes samples. (**A**) TP; (**B**) TL. **Figure S2**. XRD spectra of raw and various microbial fermentation different tobacco shapes samples. (**A**) TP; (**B**) TL. **Figure S3**. Community heat maps of raw and various microbial fermentation in TP and TL at the genus level. **Figure S4**. The cladogram showing the LAD analysis results of the bacterial community in control and fermented TP and TL revealed by LEfSe. (**A**) TP group; (**B**) TL group. **Table S1**. The standard for evaluating the sensory quality of cigarettes (QYNZY.J07.022-201). **Table S2**. Physiological characteristics of the isolate strains. **Table S3**. The sensory quality description of cigarettes in different groups. **Table S4**. The Changes of aroma components of fermented tobacco powder (TP). **Table S5**. The Changes of aroma components of fermented tobacco leaves (TL).

## Data Availability

The authors declared that the data supporting the findings of this study are available within the article and its supplementary information files.

## Abbreviations

TP: Tobacco powder
TL: Tobacco leaves
TSNA: Tobacco-specific nitrosamines
B1: *Bacillus subtilis* B1
C4: *Cytobacillus oceanisediminis* C4
CMC-Na: Carboxymethylcellulose sodium
PDA: Potato dextrose agar
PCR: Polymerase chain reactions
NCBI: National Center for Biotechnology Information
BLAST: Basic local alignment search tool
HnB: Heat-not-burn
SDE: Simultaneous distillation extraction
SEM: Scanning electron microscope
XRD: X-ray diffraction
FTIR: Fourier transform infrared spectroscopy
PCoA: Principle coordination analysis
LDA: Linear Discriminate Analysis
LEfSe: LDA effect size
C4.TP: *Cytobacillus oceanisediminis* C4.tobacco powder
C4.TL: *Cytobacillus oceanisediminis* C4.tobacco leaves
B1.TP: *Bacillus subtilis* B1.tobacco powder
B1.TL: *Bacillus subtilis* B1.tobacco leaves
NMDS: Non-metric multidimensional scaling
