# Supplementary material for: Tobacco microbial screening and application in improving the quality of tobacco in different physical states
Source: Bioresour Bioprocess. 2023 May 2;10(1):32. doi: 10.1186/s40643-023-00651-6 (PMC10992236; doi:10.1186/s40643-023-00651-6)
Supplement: Supplementary file 1 — Additional file 1: Figure S1. FTIR spectra of raw and various microbial fermentation different tobacco shapes samples. (A) TP; (B) TL. Figure S2. XRD spectra of raw and various microbial fermentation different tobacco shapes samples. (A) TP; (B) TL. Figure S3. Community heat maps of raw and various microbial fermentation in TP and TL at the genus level. Figure S4. The cladogram showing the LAD analysis results of the bacterial community in control and fermented TP and TL revealed by LEfSe. (A) TP group; (B) TL group. Table S1. The standard for evaluating the sensory quality of cigarettes (QYNZY.J07.022-201). Table S2. Physiological characteristics of the isolate strains. Table S3. The sensory quality description of cigarettes in different groups. Table S4. The Changes of aroma components of fermented tobacco powder (TP). Table S5. The Changes of aroma components of fermented tobacco leaves (TL). [file 40643_2023_651_MOESM1_ESM.pdf]

Tobacco microbial screening and application in improving the quality of tobacco in different physical states

Ying Ning[a,b], Li-Yuan Zhang[a,b], Jing Mai[b], Jia-En Su[a], Jie-Yun Cai[a], Yi Chen[a], Yong-Lei Jiang[a], Ming-Jun Zhu[b, c,] *, Bin-Bin Hu[a,] *

[a] Yunnan Academy of Tobacco Agricultural Sciences, Kunming 650021, People's Republic of China

[b] School of Biology and Biological Engineering, Guangdong Key Laboratory of Fermentation and Enzyme Engineering, South China University of Technology, Guangzhou Higher Education Mega Center, Panyu, Guangzhou 510006, People's Republic of China

[c] College of Life and Geographic Sciences, the Key Laboratory of Biological Resources and Ecology of Pamirs Plateau in Xinjiang Uygur Autonomous Region; the Key Laboratory of Ecology and Biological Resources in Yarkand Oasis at Colleges & Universities under the Department of Education of Xinjiang Uygur Autonomous Region, Kashi University, Kashi 844006, China

***Correspondence:**

Ming-Jun Zhu*

mjzhu@scut.edu.cn

Bin-Bin Hu*

hubinbin20072008@163.com

Figure. S1 FTIR spectra of raw and various microbial fermentation different tobacco shapes samples.

(A) TP; (B) TL

Figure. S2 XRD spectra of raw and various microbial fermentation different tobacco shapes samples.

(A) TP; (B) TL

Figure. S3 Community heat maps of raw and various microbial fermentation in TP and TL at the genus level

Figure. S4 The cladogram showing the LAD analysis results of the bacterial community in control and fermented TP and TL revealed by LEfSe.

(A) TP group; (B) TL group

Table S1 The standard for evaluating the sensory quality of cigarettes (QYNZY.J07.022-201)

Table S2 Physiological characteristics of the isolate strains

Table S3 The sensory quality description of cigarettes in different groups

Table S4 The Changes of aroma components of fermented tobacco powder (TP)

Table S5 The Changes of aroma components of fermented tobacco leaves (TL)

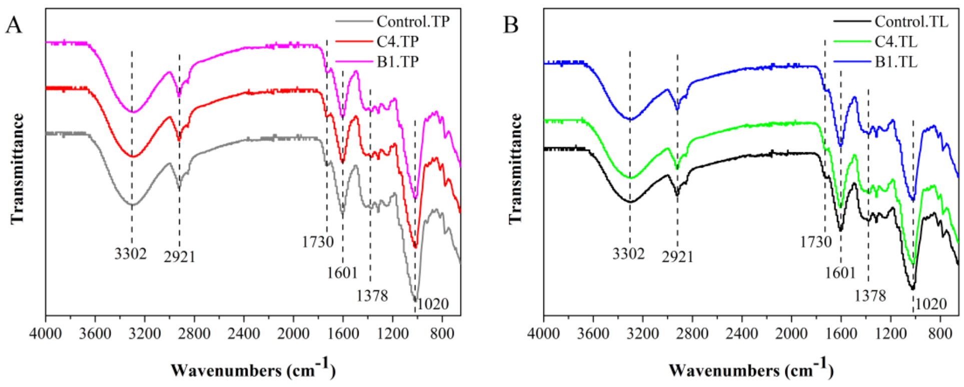

Figure. S1 FTIR spectra of raw and various microbial fermentation different tobacco shapes samples

(A)TP; (B) TL

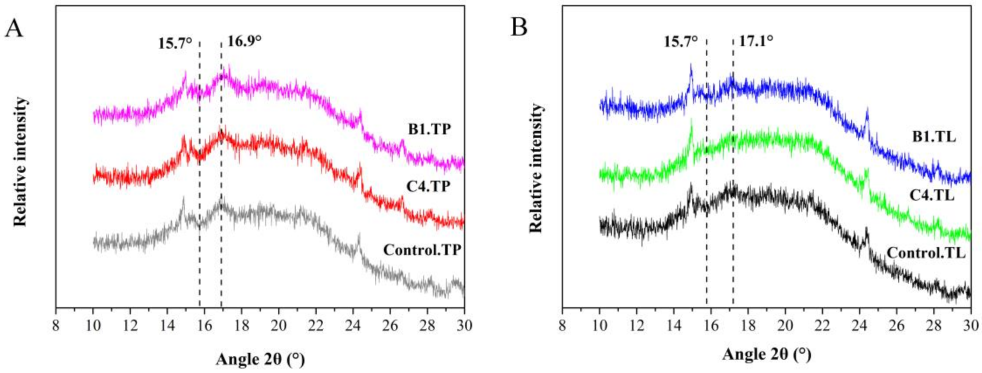

Figure. S2 XRD spectra of raw and various microbial fermentation different tobacco shapes samples

(A) TP; (B) TL

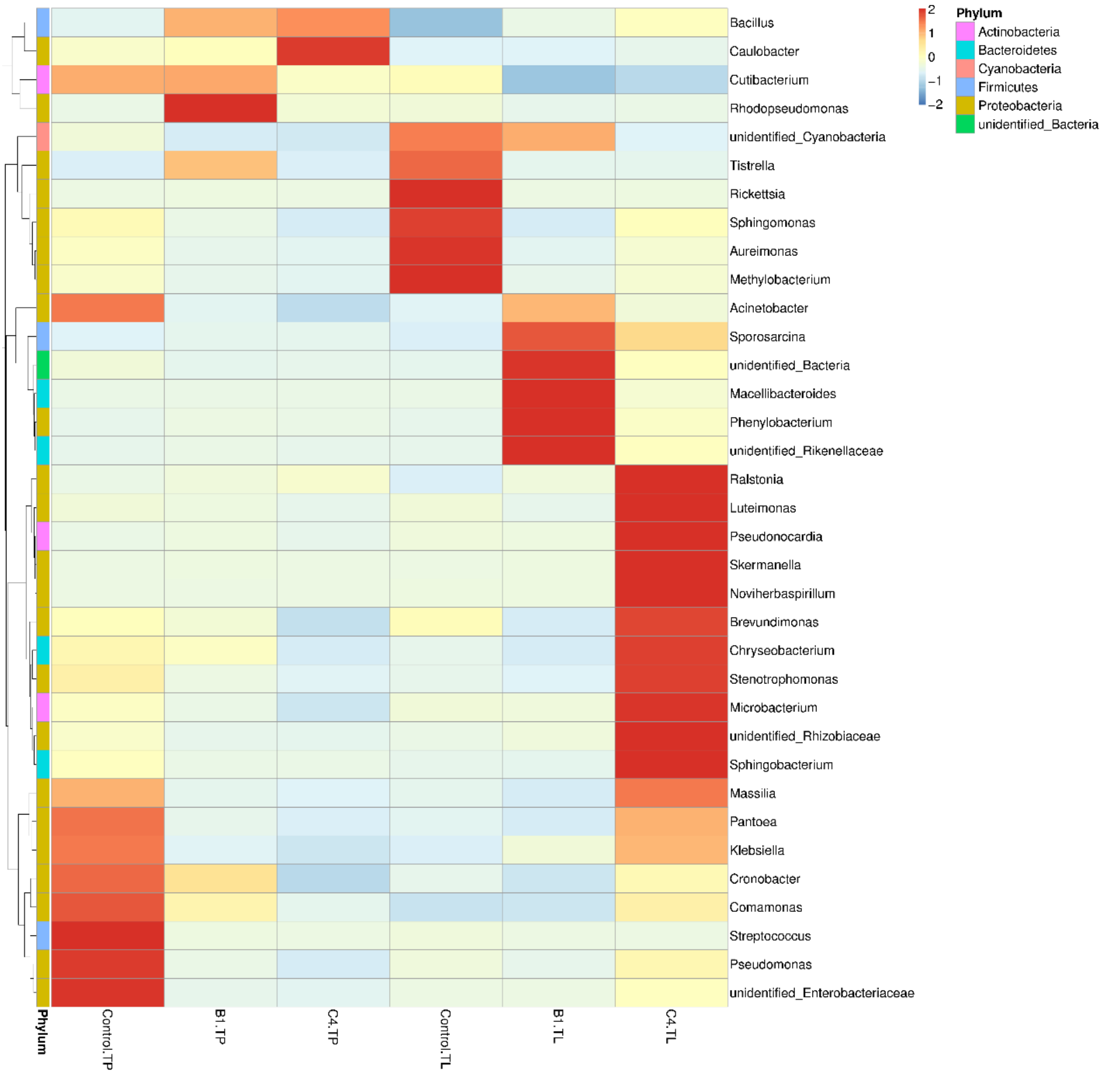

Figure. S3 Community heat maps of raw and various microbial fermentation in TP and TL at the genus level

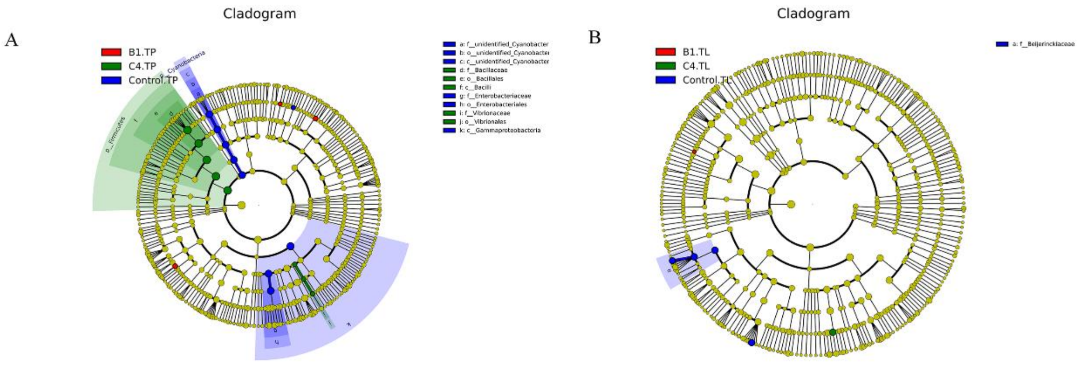

Figure. S4 The cladogram showing the LAD analysis results of the bacterial community in control and fermented TP and TL revealed by LEfSe.

(A) TP group; (B) TL group

Table S1 The **s**tandard for evaluating the sensory quality of cigarettes (QYNZY.J07.022-201)

Fractional segment	Volume of smoke		Aroma and flavor		Physiological strength		Harmony		Irritancy		Taste	
	Index	Maximum score	Index	Maximum score	Index	Maximum score	Index	Maximum score	Index	Maximum score	Index	Maximum score
I	Abundant smoke	10	Full and delicate aroma, strong pleasant sense	30	Suitable	10	Humorous	10	No stimulation	15	Comfortable	25
II	Slightly less smoke	8	Enough aroma, slightly rough, pleasant sense	25	Slightly larger or smaller	8	Relatively humorous	8	Slightly stimulation	12	Relatively comfortable	22
III	Less smoke	6	Simple and rough aroma, just pleasant sense	20	Larger or smaller	6	Just humorous	6	More stimulation	9	Just comfortable	20

Table S2 Physiological characteristics of the isolate strains

The biochemical and physiological indexes	B1	*Bacillus subtilis*[ac]	C4	*Cytobacillus oceanisediminis*[bc]
Colonial morphology	Rhabditiform	Rhabditiform	Rhabditiform	Rhabditiform
Gram staining	+	+	+	+
Sodium citrate	+	+	+	+
V.P reaction	+	+	-	-
Methyl red	-	-	+	+
Starch hydrolysis	+	+	+	+
Catalase	+	+	+	+
Nitrate reduction	+	+	+	+
pH 5.7	+	+	-	-
Growth in				
3 % NaCl	+	+	+	+
5 % NaCl	+	+	+	+
7 %NaCl	+	+	+	+

+: positive reaction; −: negative reaction.

[a] Data from Pettersson et al. (2000) (Pettersson et al. 2000).

[b] Data from Jung et al. (2016) (Jung et al. 2016) and Zhang et al. (2010) (Zhang et al. 2010).

[c] Data from the Bergey's Manual of Determinative Bacteriology and Handbook for the Identification of Common bacterial systems.

Table S3 The sensory quality description of cigarettes in different groups

Groups	Total	Sensory quality description
Control.TP	82.0^{b}	Dry and thin aroma, more delicate smoke, weak strength, and moderate volume of smoke.
C4.TP	84.0^{a}	Unique style, great coordination, rich and elegant aroma, suitable strength, good oral aroma, excellent suction feeling, significantly better than the control.
B1.TP	83.5^{a}	Slightly spicy, short and rich smoke, slight irritation, woody gas, a little messy and unclear main aroma.
Control.TL	83.0^{a}	Slightly residual, good overall, good toasted sweet.
C4.TL	83.5^{a}	Weak aroma concentration and richness, slightly sweet and sour, the back end has wood impurity, less elegant sense, the overall rhyme is consistent.
B1.TL	83.5^{a}	Good aroma richness, poor coordination, weak physiological strength, slightly good sweet rhyme, and slightly low skeleton sense, physiological strength increasingly improves during smoking.

Notes: Values are means ± standard deviations (n=3).

Different letters (a, b) show statistically significant differences within each tobacco powder/tobacco leaves group ($p < 0.05$), and the same letter indicates no significant difference.

Table S4 The Changes of aroma components of fermented tobacco powder (TP)

Types	Components (μg/g)	Control.TP	C4.TP	B1.TP
Plastid pigments	Neophytadiene	316.78	567.63	456.17
	β-Damascenone	3.09	30.32	27.26
	Megastigmatrienone B	6.47	23.52	24.31
	Dihydroactinidiolide	0.75	17.35	15.99
	Geranyl acetone	7.15	14.91	14.72
	Megastigmatrienone D	5.67	12.66	4.73
	Megastigmatrienone C	7.31	8.01	10.61
	Megastigmatrienone A	2.1	3.94	6.17
	β-Damascone	0.6	2.69	2.82
	4-Oxoisophorone	0.1	0.86	0.29
	Damascenone	0.29	0.46	0.71
	Total	350.31	682.35	563.78
Ceberoids	Solanone	23.2	64.95	59.29
	Cembrenediol 3	4.01	15.37	18.76
	Cembrenediol 4	8.32	8.11	46.74
	Cembrenediol 2	1.38	3.22	1.95
	Cembrenediol 1	1.80	1.83	2.10
	Total	38.71	93.48	128.84
Phenylalanines	Dibutyl phthalate	11.77	42.27	40.40
	Phenyl ethanol	0.27	9.41	8.66
	Benzyl alcohol	0.06	7.53	7.55
	4-Vinyl-2-Methoxyphenol	0.10	0.22	0.26
	Phenylacetaldehyde	0.11	0.07	0.21
	Total	12.31	59.5	57.08
Maillard reaction products	2, 3-dihydro2-methoxybenzofuran	0.31	2.74	2.22
	3-Methoxybenzaldehyde	0.55	2.27	2.31
	4-diethylaminobenzaldoxime	0.11	1.12	1.30
	2,4-Heptadienal	0.35	0.22	0.31
	Pyrrole	0.09	0.15	0.14
	Phenylacetaldehyde	0.11	0.07	0.21
	Furfural	0.36	0.01	0.01
	Total	1.88	6.58	6.50
Nicotinoids	Myosmine	0.21	0.33	1.22
	Total	0.21	0.33	1.22
Others	Total	142.09	405.51	348.40

Table S5 The Changes of aroma components of fermented tobacco powder (TL)

Types	Components (μg/g)	Control .TL	C4.TL	B1.TL
Plastid pigments	Neophytadiene	586.23	979.67	1144.25
	β-Damascenone	0.57	2.90	2.99
	Megastigmatrienone B	6.53	38.23	50.35
	Dihydroactinidiolide	2.03	10.56	9.79
	Geranyl acetone	2.47	5.55	3.91
	Megastigmatrienone D	5.87	35.11	41.09
	Megastigmatrienone C	0.60	3.54	5.83
	Megastigmatrienone A	1.26	7.59	10.04
	β-Damascone	10.68	29.52	38.07
	4-Oxoisophorone	0.21	0.53	0.79
	Damascenone	0.20	0.38	0.79
	Total	616.65	1113.58	1307.90
Ceberoids	Solanone	0.34	27.67	46.91
	Cembrenediol 3	2.06	4.82	7.41
	Cembrenediol 4	6.15	4.91	2.60
	Cembrenediol 2	1.79	4.34	8.96
	Cembrenediol 1	0.90	1.80	6.33
	Total	11.24	43.54	72.21
Phenylalanines	Dibutyl phthalate	1.62	38.55	34.13
	Phenyl ethanol	4.82	7.29	12.84
	Benzyl alcohol	6.45	0.05	22.95
	4-Vinyl-2-Methoxyphenol	2.43	1.64	0.16
	Phenylacetaldehyde	3.07	7.18	0.35
	Total	18.39	54.71	70.43
Maillard reaction products	2, 3-dihydro2-methoxybenzofuran	0.31	1.30	0.54
	3-Methoxybenzaldehyde	0.37	0.34	0.92
	4-diethylaminobenzaldoxime	0.15	0.98	0.91
	2,4-Heptadienal	0.16	0.24	0.26
	Pyrrole	0.10	0.09	0.21
	Phenylacetaldehyde	3.07	7.18	0.35
	Furfural	3.29	0.08	12.74
	Total	7.45	10.21	15.93
Nicotinoids	Myosmine	0.42	0.28	0.91
	Total	0.42	0.28	0.91
Others	Total	50.99	243.01	270.95

References

Jung J, Jeong H, Kim HJ, Lee D-W, Lee SJ (2016) Complete genome sequence of *Bacillus oceanisediminis* 2691, a reservoir of heavy-metal resistance genes. Mar Genomics 30:73-76 doi:10.1016/j.margen.2016.07.002

Pettersson B, de Silva SK, Uhlén M, Priest FG (2000) *Bacillus siralis sp*. nov., a novel species from silage with a higher order structural attribute in the 16S rRNA genes. Int J Syst Evol Microbiol 50(6):2181-2187 doi:10.1099/00207713-50-6-2181

Zhang J, Wang J, Fang C, Song F, Xin Y, Qu L, Ding K (2010) *Bacillus oceanisediminis sp*. nov., isolated from marine sediment. Int J Syst Evol Microbiol 60(12):2924-2929 doi:10.1099/ijs.0.019851-0
